# Supplementary material for: Experimental study of water migration characteristics in compacted loess subjected to rainfall infiltration
Source: PLoS One. 2022 Sep 9;17(9):e0274265. doi: 10.1371/journal.pone.0274265 (PMC9462739; doi:10.1371/journal.pone.0274265)
Supplement: Attachment — Submitted filename: review(PONE-D-22-12253).docx [file pone.0274265.s001.docx]

In this paper, the water migration characteristics of compacted loess under different rainfall conditions were studied, and the corresponding results were obtained. However, there are still some confusions about the article, as follows. I recommend that the article should be published after a substantial revision.

1. Introduction: Authors cite some papers here for literature review. However, most of the text is a brief introduction to these studies and the generalization and summary of these studies are relatively lacking. In this case, the necessity of the article is unconvincing. Authors are advised to make a substantial revision of the Introduction, systematically review of previous research and highlight the innovation are necessary.
2. Line 71: ‘were’ not ‘was’
3. Fig. 1: Figure b should also add a corresponding legend description.
4. Fig. 2: ‘processes’ not ‘process’
5. Line 149: The latitude and longitude coordinates of the sampling point need to be given.
6. Line 155: Please explain the reasons for these three dry density choices.
7. Eq. 5 and 6: The format is different from other formulas
8. Authors shows a lot of results in the results section, but some phenomena are not further explained. E.g., line 223-229: No further analysis on conductivity changes is given here.
9. As the authors say, understanding the water transport properties of loess has important practical significance. In the discussion, however, I did not find the authors to make any practical recommendations based on the research results.
